# Phenotypic and cytogenetic variability of patau syndrome in Morocco

**DOI:** 10.4314/ahs.v23i4.60

**Published:** 2023-12

**Authors:** Hanane Ait hammou, Mariam Sennaoui, Fatimazahra Bouzid, Kenza Dafir, Meriem El Qabli, Hassan Akallakh, Maria Mansouri, Fadl Mrabih Rabou Maoulainine, Mohammed Bouskraoui, Nisrine Aboussair

**Affiliations:** 1 Department of Genetics, Clinical Research Center, Mohammed VI University Hospital Center of Marrakech, Morocco; 2 Faculty of Medicine and Pharmacy, University of Cadi Ayyad, Marrakech, Morocco; 3 Neonatal Intensive Care Unit, Mother and Child Hospital, Mohammed VI University Hospital Center of Marrakech, Morocco; 4 Pediatric A department, Mother and Child Hospital, Mohammed VI University Hospital, Center of Marrakech, Morocco

**Keywords:** Patau syndrome, Genetic counseling of patau syndrome, Cytogenetics

## Abstract

The objective of this work was to identify phenotypic features and cytogenetic aspects of trisomy 13 in Moroccan population. The retrospective study was conducted on a group of 9 cases diagnosed cytogenetically with trisomy 13.

The study of sex ratio showed a slight female dominance in our group of cases. The major clinical findings included: Holoprosencephaly, microphthalmia and anophthalmia, coloboma of iris, cleft lip and palate, nasal and ear abnormalities, retrognathism and sloping forehead, polydactyly, capillary hemangiomas, omphalocele, congenital heart defect, renal abnormalities, cryptorchidism, language delay.

The cytogenetic study showed the dominance of the free and homogeneous trisomy 13 (56%). Patients who have this formula are dead at an early age (does not exceed one month). However, each of the chromosomal formula, trisomy 13 by translocation and partial trisomy 13 t (13;18), was found in 20% of our patients.

The partial trisomy 13 t (13;18) is the only variant that is still alive and the patients with this anomaly suffer mainly from renal and cardiac anomalies with slight dysmorphia and psychomotor retardation.

Our study shows the interest of the cytogenetic analysis in the diagnosis accuracy and in the genetic counseling of patients with Patau syndrome and their parents.

## Introduction

Trisomy 13 or Patau syndrome (PS) is a chromosomal abnormality caused by the presence of an extra 13 chromosome. This syndrome is the rarest of the trisomies that can lead to a full-term birth of a living child (Savva et al., 2010), it is also the most frequent chromosomal abnormality which is characterized by severe clinical picture of multiple congenital anomalies and which leaves little hope of survival after his diagnosis (Plaiasu et al., 2010). The pathology was first described by Patau et al. (Patau et al., 1960) and since then more than 100 different abnormalities have been described (Jones, 2006)

It is characterized by the association of cerebral malformations (holoprosencephaly), facial dysmorphia, ocular abnormalities, postaxial polydactyly, visceral malformations and very severe psychomotor retardation. Its incidence is estimated between 1/8000 and 1/15000 births and more than 95% of affected fetuses die in utero.

In the present work, we report a series of 9 patients with PS collected at the genetics department of the Mohammed VI University Hospital in Marrakech. Our objective was to (1) determine the different clinical features and cytogenetic aspects of PS in Morocco and (2) study the correlation between the different clinical signs and the cytogenetic aspects of PS.

## Patients and methods

This study is a retrospective clinical and cytogenetic one conducted on a group of 9 cases with trisomy 13 between 2008 and 2019. These patients were referred to the genetic department of Mohammed VI University Hospital Center of Marrakech for a variable dysmorphic and polymalformative syndrome.

Clinical examination and cytogenetic analysis of these patients allowed us to confirm the diagnosis of Patau syndrome. The cytogenetic technique used in this study is the constitutional R-banded karyotype carried out for all patients from T lymphocytes cultured in vitro and blocked in metaphase.

## Results

**Figure 1:**
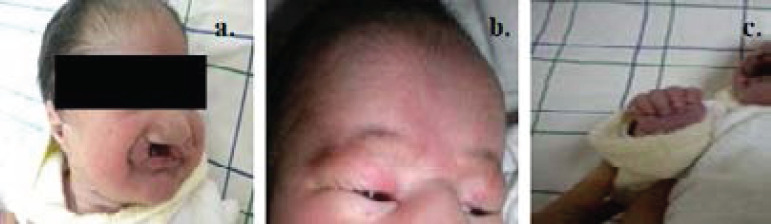
Some abnormalities observed among patients of the sample: (a) Bilateral cleft lip and cleft palate, (b) microphthalmia and hemangioma, (c) postaxial polydactyly of the right hand

The dysmorphological examination and the malformative assessment (Fig.1) allowed determining the different clinical elements of our cases with PS.

Table 1 summarizes the clinical observations of these patients. The main finding observed in our group of patients consisted of holoprosencephaly, microphthalmia/anophthalmia, coloboma of iris, cleft lip/cleft palate, sloping Forehead, retrognathism, nasal anomalies, ear anomalies, polydactyly (Hands/Feet), capillary hemangiomas, omphalocele, congenital heart defect, renal abnormalities, genitalia abnormalities, psychomotor retardation.

**Table 1:** Clinical features of patients

Clinical sign	C1	C2	C3	C4	C5	C6	C7	C8	C9
Holoprosencephaly	-				-	+	-		-
Microphtalmia/anophthalmia	+	+	-	-	-	+	-	-	-
Coloboma of iris	-	+		-	-	-	-	-	-
cleft lip/cleft palate	+	+	+	+	-	-	+	+	-
Sloping Forehead	+	-	-	+	-	+	-	-	-
Retrognatism	+	-	-	-	-	+	-	-	-
Nasal anomalies	-	-	-	+	-	+	+	-	-
Ear anomalies	+	-	-	+	+	+	-	+	+
Polydactyly (Hands/Feet)	+	+	+	-	-	+	+	+	+
Capillary hemangiomas	-	+	-	+	+	-	-	+	+
Omphalocele	-	-	-	-	-	-	-	+	-
Congenital heart defects	-		+		+	+	+		+
Renal abnormalities					+				+
Genitalia abnormalities	-	-	-	+	-	-	-	-	-
Psychomotor retardation					+				+

**Figure 2:**
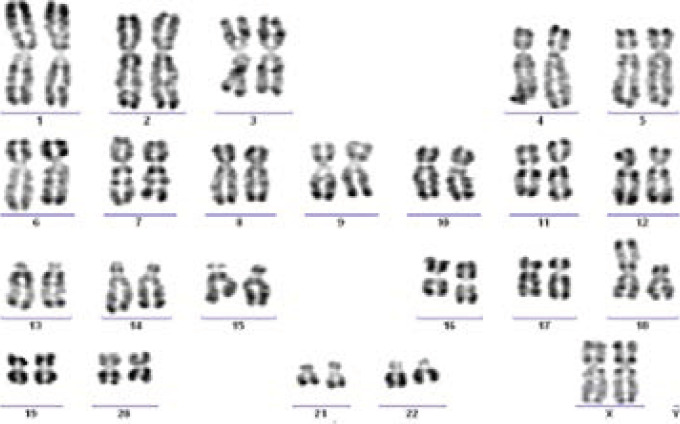
Karyotype of partial trisomy 13 with t (13;18)

Clinical diagnosis was confirmed by chromosomal analysis: 5 cases of free and homogeneous trisomy 13 (cases 1, 2, 4, 6 and 8), 2 of trisomy 13 by Robertsonian translocation (cases 3 and 7) and two cases of trisomy of the long arm of chromosome 13 secondary to a translocation (13;18) of paternal origin (Fig.2) (cases 5 and 9) (table 2). Parental karyotypes were normal in case 3, indicating a de novo translocation. However, no data about the parents were available in case 7.

**Table 2:** Karyotype results for the 9 cases

Cases	Age	Gender	chromosomal formula	Origin
1	1 month	female	47, XX, +13	De novo
2	9 days	male	47, XY, +13	De novo
3	1 day	female	46, XX, +13, rob (13;13) (q10; q10)	De novo
4	12 days	male	47, XY, +13	De novo
5	8 years	female	46,XX,der(18)t(13;18)(q14;p 11)pat	Paternal origin
6	1 month	male	47, XY, +13	De novo
7	12 days	female	46, XX, +13, rob (13;14) (q10; q10)	?
8	3 days	male	47, XY, +13	De novo
9	2 years	female	46,XX,der(18)t(13;18)(q14;p 11)pat	Paternal origin

## Discussion

### Analysis of clinical features

The Patau syndrome causes serious physical and mental abnormalities. The incidence of this syndrome is approximately one per 12,000 live births (Baty et al., 1994; Delatycki and Garder, 1997). Clinical diagnosis is based on suggestive signs and symptoms. However, the variability of disease expression can sometimes complicate the diagnostic process. In the present study, we tried to identify the different variable elements of PS in Morocco.

The study of sex ratio showed a slight female dominance in our of cases (sexe ratio: 1,25), which is discordant with several studies of literature (Alberma, et al., 2012; Bugge *et al.*, 2007; Huether et al., 1996). This can be explained by the small size of group of patients studied.

The most severe abnormality is holoprosencephaly which is the most common forebrain defect in humans, with a prevalence of 1/250 in embryos and around 1 / 10,000 in infants born alive (Edison et al., 2003; Leoncini et al., 2008). This malformation causes significant anomalies associated with the face. Children with the most severe forms only live a few months.

In our study group, theses abnormalities were detected only in one case (1/4). Unfortunately, the radiological examination was not performed for four patients due to death at an early age. Microphthalmia and anophthalmia are birth defects of a baby's eye(s), affecting successively about 14/100000 new borns and 3/100000 neonates (Morrison et al., 2002). This abnormality iis found in 60%-70% of Patau syndrome's cases (Firth et al., 2005; Morrison et al., 2002). However, in the present study, this abnormality is present in 33% of cases. This contradiction can be explained by the small size of cases group studied. Moreover, coloboma of iris is present in one case of our cases group (11%), which is associated with microphthalmia.

Cleft lip and palate are among the most common birth defects. The frequency of this anomaly in PS is between 60% and 70% (Stoll et al., 1991; Milerad et al., 1997). In our study, we found a frequency of 67%, which is consistent with literature.

Nasal and ear abnormalities are also common in PS. It was detected successively in 33% and 66% of our group of cases. In one patient (C6), the nasal abnormality was associated with holoprosencephaly, who present a broad nasal root. The most abnormalities forms of the ear found in this study are faun ears and the absence of ear lobes.

Retrognatism and sloping forehead present as a birth defect, which are also found in our cases group with successively a frequency of 22% and 33%.

Polydactyly (Hands/Feet) is found in 60%-70% of cases with PS (Firth et al., 2005). In our study, this abnormality presents a very high frequency of 77% (5 cases of hand polydactyly and one case of foot polydactyly).

As for dermatological defects, capillary hemangiomas are the most common and have been described in up to 56% of cases (Taylor, 1986). The frequency of these anomalies in our study is 55%, which is consistent with the literature.

Omphalocele is a rare abdominal wall defect that occurs in 1 in 4,000 births (CDC, 2020) and is associated with a high rate of mortality (25%) and severe malformations.

In our study, only one case of omphalocele (type 1) has been found (11%), which is characterized by a parietal defect less than 2,5cm and the absence of liver.

Congenital heart defect is among the most common abnormalities visualized in PS, being found in 80% of cases (Cassidy and Allanson, 2010; Firth et al., 2005). In our study, we have identified this abnormality in just 55% of patients. Atrial septal defect (ASD), ventricular septal defect (VSD) and persistent ductus arteriosus (PDA) are generally the anomalies detected in this work.

Renal abnormalities are present in about 50 to 60% of affected individuals (Egli et al., 1973, Warkany et al., 1966). This anomaly is found in 2 cases of our group (22%) who present renal hypoplasia.

According to Petry et al. (Petry et al., 2013), genitalia abnormalities are more common among males than females and mainly represented by cryptorchidism and micropenis. In the present study, only one case with cryptorchidism that was detected.

Psychomotor retardation is also common in patients with PS, which include slowed speech, decreased movement and impaired cognitive function. 2 cases in our group present a language delay and a start walking from 3 years old.

### Study of cytogenetic aspects

Cytogenetically, our results showed 5 cases of free homogeneous trisomy 13 (56%), 2 cases of complete trisomy 13 by Robertsonian translocation (20%) and 2 cases of partial trisomy of the long arm of chromosome 13 secondary to a reciprocal translocation (13;18) of paternal origin (20%). This data consistent with the literature where the frequency of the variant free homogeneous is the most important (approximately 75%) (Firth et al., 2005). However, in 20% of cases, it is a Robertsonian translocation, with the supernumerary chromosome13 being attached on another acrocentric chromosome (chromosomes 13, 14, 15, 21 or 22) (Firth et al., 2005). Rarely, trisomy 13 is due to a reciprocal translocation between 13 and a non-acrocentric chromosome.

### Correlation genotype-phenotype

According to our study, the free and homogeneous trisomy 13 variant (cases 1, 2, 4, 6 and 8) was the most severe, which includes almost all the anomalies mentioned in the clinical picture of PS. Patients who have this variant are dead at an early age that does not exceed one month. Concerning partial trisomy 13 by reciprocal translocation (case 3 and case 7), patients with this variant don't present all the abnormalities that are found in free and homogeneous trisomy 13 variant, but generally: cleft palate, nose anomalies, polydactyly and congenital heart defects.

As for partial trisomy 13 t (13;18) which is the only variant that can survive (Verloes, 2008), the two patients (case 5 and 9, who have successively 8 and 4 years) suffer mainly from renal and cardiac anomalies with slight dysmorphia and psychomotor retardation.

### Genetic counseling

The recurrence risk for free trisomy 13 variant is low (<1%). In this situation, the parental karyotype is not necessary because most cases of this variant are an isolated occurrence. So, the genetic counseling is reassuring for parents of cases 1, 2, 4, 6 and 8. In fact, the main factor of occurrence of this anomaly may be ovular aging related to maternal age. Then, majority of maternal aneuploidies are caused by de novo segregation error during the first meiotic division (Hassold et al., 1995; Terret and Wassmann, 2008).

For the cases 1, 2 and 4, maternal age was between 39 and 45 years. However, for the cases 6 and 8, maternal age was between 27 and 30 years. Therefore, for these last two cases, maternal age did not represent a risk for the appearance of trisomy 13.

Concerning the other two variants (Robertsonian translocations and partial trisomy 13), the risk of recurrence is high and parental karyotype must be performed. In this situation, if thethe parents' karyotype is normal (case 3), this is de novo translocation and therefore genetic counseling is reassuring. However, if a balanced translocation is found in one of the parents (case 5 and 9), there will be a risk of recurrence of trisomy 13 in the siblings by meiotic malsegregation of the parental translocation. Generally, the risk varies depending on the chromosome involved, length of the segment and the sex of parent carrying the balanced anomaly (Gardner and Sutherland, 1996). In our case, the father being a carrier balanced chromosomal rearrangement (for the two cases 5 and 9), then the risk of rebirth of a child with the chromosomal imbalance is 25%. Moreover, 25% to have a normal child and 25% to have a child with a balanced translocation. Therefore, prenatal cytogenetic diagnosis should be advised for subsequent pregnancies.

Surgical treatment of malformations does not significantly alter the prognosis. Trisomy 13 is very severe; half of children die in the first month and 90% before 1 year from cardiac, renal or neurological complications. Prolonged survival (sometimes until adulthood) is however possible, in particular in cases of mosaicism, partial trisomy (cases 5 and 9) and if there is no major brain malformation.

## Conclusion

This study demonstrates the importance of cytogenetic tests for the diagnosis of Patau syndrome. In fact, the diagnosis of this syndrome is only possible using clinical examination and cytogenetic tools. Prenatal cytogenetic testing is necessary for genetic counseling because the chromosome analysis of patients and their parents can prevent the birth of another child with this syndrome.

## References

[1] Alberman E, Mutton D, Morris JK (2012). Cytological and epidemiological findings in trisomies 13, 18, and 21: England and Wales 2004-2009. American Journal of Medical Genetics Part A.

[2] Baty BJ, Brent L, Cary JC (1994). Natural history of trisomy 18 and trisomy 13, I: growth, physical assessment, medical histories, survival, and recurrence risk. American Journal of Medical Genetics.

[3] Bugge M, Collins A, Hertz JM (2007). Non-disjunction of chromosome 13. Human molecular genetics.

[4] Cassidy SB, Allanson JE (2010). Management of genetic syndromes.

[5] CDC (2020). Facts about Omphalocele.

[6] Delatycki M, Garder R (1997). Three cases of trisomy 13 mosaicism and a review of the literature. Clinical Genetics.

[7] Edison R, Muenke M (2003). The interplay of genetic and environmental factors in craniofacial morphogenesis: holoprosencephaly and the role of cholesterol. Congenital Anomalies (Kyoto).

[8] Egli F, Stalder G (1973). Malformations of kidney and urinary tract in common chromosomal aberrations. I. Clinical studies. Humangenetik.

[9] Firth HV, Hurst JA (2005). Oxford Desk Reference: Clinical Genetics and Genomics.

[10] Gardner RJM, Sutherland GR (1996). Inversion. Chromosome abnormalities and genetic counselling.

[11] Hassold T, Merrill M, Adkins K (1995). Recombinaison and maternal age-dependent non- disjunction: Molecular studies of trisomy 16. American Journal of Human Genetic.

[12] Heuther CA, Martin RM, Stoppelman S M (1996). Sex ratios in fetuses and liveborn infants with autosomal aneuploidy. American journal of medical genetics.

[13] Jones KL (2006). Smith's recognizable patterns of human malformation 6th.

[14] Leoncini E, Baranello G, Orioli IM, Annerén G, Bakker M, Bianchi F, Bower C, Canfield MA, Castilla EE, Cocchi G, Correa A, De Vigan C, Doray B, Feldkamp ML (2008). Frequency of holoprosencephaly in the International Clearinghouse Birth Defects Surveillance Systems: searching for population variations. Birth defects research. Part A, Clinical and molecular teratology.

[15] Milerad J, Larson O, Hagberg C, Ideberg M (1997). Associated malformations in infants with cleft lip and palate: a prospective, population-based study. Pediatrics.

[16] Morrison D, FitzPatrick D, Hanson I, Williamson K, van Heyningen V, Fleck B, Jones I, Chalmers J, Campbell H (2002). National study of microphthalmia, anophthalmia, and coloboma (MAC) in Scotland: investigation of genetic aetiology. Medical Genetics.

[17] Plaiasu V, Ochiana D, Motei G, Anca I, Georgescu A (2010). Clinical relevance of cytogenetics to pediatric practice. Postnatal findings of Patau syndrome - Review of 5 cases. Maedica (Buchar).

[18] Patau K, Smith DW, Therman E, Inhorn SI (1960). Multiple congenital anomaly caused by an extra autosome. The lancet.

[19] Petry P, Polli JB, Mattos VF (2013). Clinical features and prognosis of a sample of patients with trisomy 13 (Patau syndrome) from Brazil. American Journal of Medical Genetics Part A.

[20] Savva GM, Walker K, Morris JK (2010). The maternal age-specific live birh prevalence of trisomies 13 and 18 compared to trisomy 21 (Down syndrome) ». Prenatal Diagnosis.

[21] Stoll C, Alembik Y, Dott B, Roth M (1991). P. Epidemiological and genetic study in 207 cases of oral clefts in Alsace, north-eastern France. Journal of medical genetics.

[22] Terret E, Wassmann K (2008). Le point faible méiotique : la première division. Médecine Sciences (Paris).

[23] Verloes A (2008). Trisomy 13. Rare diseases.

[24] Warkany J, Passarge E, Smith LR (1966). Congenital malformations in autosomal trisomy syndrome. American Journal of Diseases of Children.

